# The *Immp2l* mutation causes age‐dependent degeneration of cerebellar granule neurons prevented by antioxidant treatment

**DOI:** 10.1111/acel.12426

**Published:** 2015-11-30

**Authors:** Chunlian Liu, Xue Li, Baisong Lu

**Affiliations:** ^1^ Department of Center for Reproductive MedicineGeneral HospitalNingxia Medical UniversityNingxia750004China; ^2^ Key Laboratory of Fertility Preservation and Maintenance of Ministry of EducationNingxia Medical UniversityNingxia750004China; ^3^ Wake Forest University Health SciencesInstitute for Regenerative MedicineWinston‐SalemNC27157USA; ^4^ Department of PathologyBeijing Chao‐Yang HospitalCapital Medical UniversityBeijingChina

**Keywords:** aging, ataxia, cerebellum neurodegeneration, *Immp2l*, mice, reactive oxygen species

## Abstract

Reactive oxygen species are implicated in age‐associated neurodegeneration, although direct *in vivo* evidence is lacking. We recently showed that mice with a mutation in the *Inner Mitochondrial Membrane Peptidase 2‐like* (*Immp2l*) gene had elevated levels of mitochondrial superoxide, impaired fertility and age‐associated phenotypes, including kyphosis and ataxia. Here we show that ataxia and cerebellar hypoplasia occur in old mutant mice (> 16 months). Cerebellar granule neurons (CGNs) are significantly underrepresented; Purkinje cells and cells in the molecular layer are not affected. Treating mutant mice with the mitochondria‐targeted antioxidant SkQ1 from 6 weeks to 21 months protected cerebellar granule neurons. Apoptotic granule neurons were observed in mutant mice but not in age‐matched normal control mice or SkQ1‐treated mice. Old mutant mice showed increased serum protein carbonyl content, cerebellar 4‐hydroxynonenal (HNE), and nitrotyrosine modification compared to old normal control mice. SOD2 expression was increased in Purkinje cells but decreased in granule neurons of old mutant mice. Mitochondrial marker protein VDAC1 also was decreased in CGNs of old mutant mice, suggesting decreased mitochondrial number. SkQ1 treatment decreased HNE and nitrotyrosine modification, and restored SOD2 and VDAC1 expression in CGNs of old mutant mice. Neuronal expression of nitric oxide synthase was increased in cerebella of young mutant mice but decreased in old mutant mice. Our work provides evidence for a causal role of oxidative stress in neurodegeneration of *Immp2l* mutant mice. The *Immp2l* mutant mouse model could be valuable in elucidating the role of oxidative stress in age‐associated neurodegeneration.

## Introduction

Cerebellar granule neurons (CGNs) are affected in several human diseases. In Cockayne syndrome, a human hereditary DNA repair disorder, CGNs degenerate and are reduced in number (Kohji *et al*., 1998). CGN loss also occurs in many prion disease cases (Collinge *et al*., 1992; Faucheux *et al*., 2011) and in transgenic mice expressing a prion protein with insertional mutation (Chiesa *et al*., 2000). Many drugs are associated with drug‐induced cerebellar ataxia (van Gaalen *et al*., 2014). Although the neuropathology behind this effect is unclear, several neurotoxic substances (e.g., methyl chloride, methyl bromide, thiophene, ethanol, and 2‐chloropropionic acid) cause granule cell degeneration in adult animals (Tavares & Paula‐Barbosa, 1982; Fonnum & Lock, 2000). Granule neuron loss is also observed in a mouse model of Friedreich's ataxia (Simon *et al*., 2004). However, it is unclear why CGN deteriorate in these situations.

Oxidative stress from mitochondrial dysfunction has been implicated in age‐associated neurodegenerative diseases such as Alzheimer's disease (Belkacemi & Ramassamy, 2012; von Bernhardi & Eugenin, 2012), Parkinson's disease (Zhou *et al*., 2008; Hauser & Hastings, 2013), Huntington's disease (Stack *et al*., 2008; Johri & Beal, 2012), amyotrophic lateral sclerosis (Mancuso *et al*., 2006; Barber & Shaw, 2010), and prion diseases (Brown, 2005; Haigh *et al*., 2011). Oxidative stress is also implicated in CGN degeneration caused by toxic substances (Fonnum & Lock, 2004). However, *in vivo* evidence that oxidative stress causes CGN deterioration is lacking.

Many mouse models show CGN degeneration during development. In Weaver mice, granule neurons die of apoptosis before they reach the granule layer (Rakic & Sidman, 1973). *Leaner* mice lose granule neurons at about postnatal day 10 (Herrup & Wilczynski, 1982). *Staggerer* mice, *lurcher* mice (*Grid2* mutant), Purkinje cell degeneration mice, and astroglial Dicer knockout mice lose CGN secondary to Purkinje cell or astroglial abnormality (Wetts & Herrup, 1982; Herrup & Sunter, 1987; Wang & Morgan, 2007). Although these models are useful for studying cerebellar neurodevelopment, they are not suitable for studying age‐associated neurodegeneration. Similarly, although harlequin mice with reduced AIF1 expression show CGN deficiency at 3–4 months (Klein *et al*., 2002), this age is still too young to mimic neurodegeneration during aging.

Inner Mitochondrial Membrane Peptidase 2‐like (IMMP2L) is a peptidase located on the mitochondrial inner membrane. It cleaves the space‐sorting signal peptide sequences of cytochrome c1 (CYC1) and mitochondrial glycerol phosphate dehydrogenase 2 (GPD2), the two known substrates for IMMP2L. We reported that mutation of both *Immp2l* alleles impaired processing of CYC1 and GPD2 signal peptide sequences in mice (Lu *et al*., 2008). Isolated mitochondrial from *Immp2l* ^*−/−*^ mice produced superoxide at an increased rate (Lu *et al*., 2008), but showed normal GPD2 and mitochondrial complex III activities (CYC1 is a subunit of complex III) and mitochondrial bioenergetic capacity (Bharadwaj *et al*., 2014).

Although their lifespans are not reduced compared to normal control mice, *Immp2l* ^*−/−*^ mice show erectile dysfunction, defective oogenesis (Lu *et al*., 2008), reduced food intake (Han *et al*., 2013), bladder dysfunction (Soler *et al*., 2010), early onset of ataxia and kyphosis (George *et al*., 2011), and age‐dependent spermatogenic damage (Lu *et al*., 2008; George *et al*., 2012). We propose that these abnormal phenotypes in *Immp2l* ^*−/−*^ mice could result from two major effects of elevated mitochondrial superoxide production: negation of the signaling molecule nitric oxide (NO), and increased formation of other forms of reactive oxygen species. Following our observation of ataxia in *Immp2l* ^*−/−*^ mice older than 16 months (George *et al*., 2011), this manuscript reports age‐dependent CGN degeneration in old *Immp2l* ^*−/−*^ mice, and the protective effects of the antioxidant SkQ1 on CGN degeneration. Our data suggest that the *Immp2l* ^*−/−*^ mouse is a novel model in which to examine the role of oxidative stress in age‐associated neurodegeneration.

## Results

### Immp2l mutant mice show CGN loss after the age of 16 months

**Figure 1:**
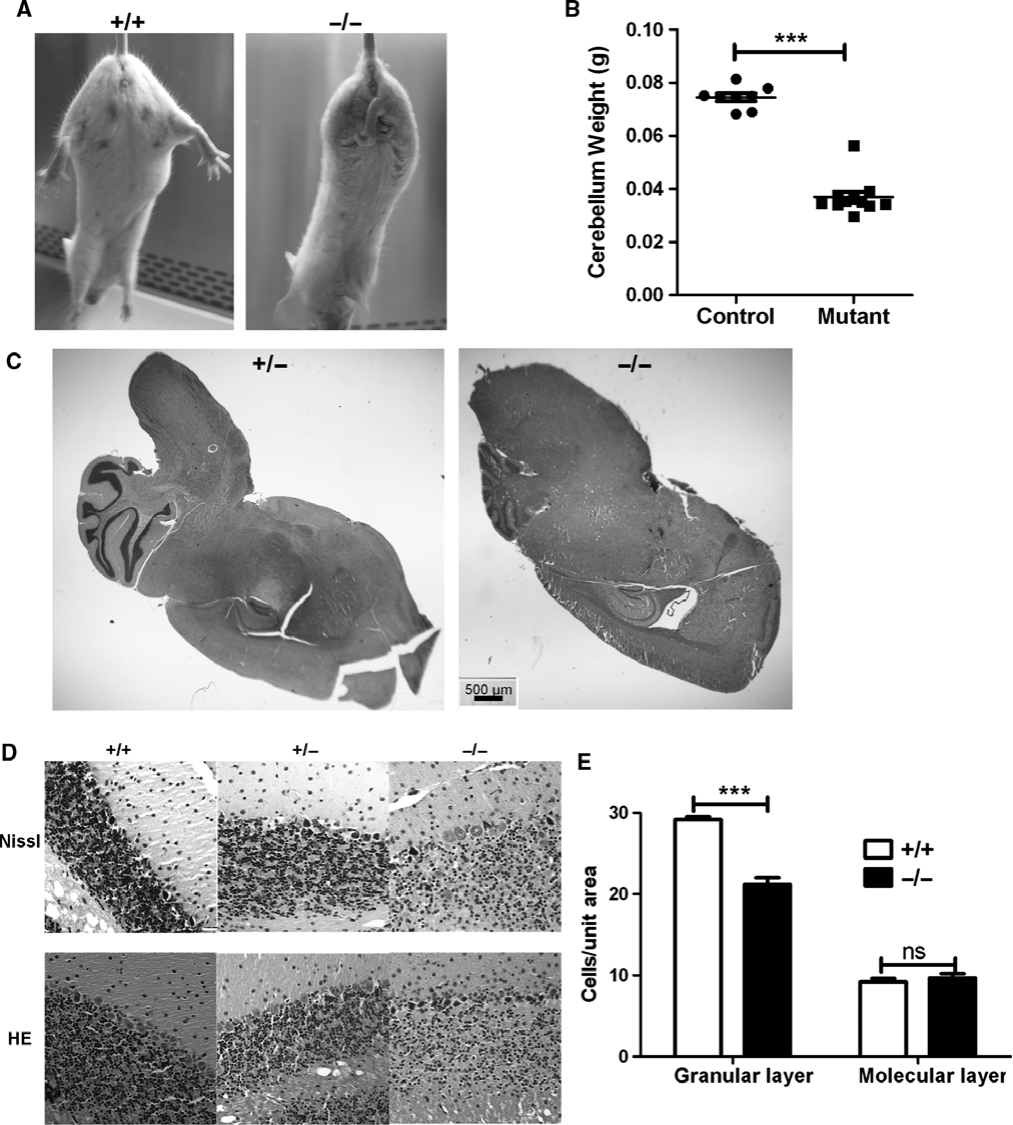
Cerebellar granule neuron loss in old mutant mice. (A) Clasp response after tail suspension. When suspended by their tails and slowly lowered, normal control mice extend their hind legs, but mutant mice clasp their hind legs to their bodies. Mice were tested at about 20 months old. (B) Reduced cerebellar weight of old mutant mice vs. controls; mice were 19–25 months old when necropsied. Each point indicates one animal. Control mice included 3+/+ and 5+/− mice. ***Indicates *P* < 0.0001 by *t*‐test. (C) H&E staining of sagittal brain sections indicating smaller cerebellar size of mutant mice. (D) Nissl (top) and hematoxylin and eosin (HE; bottom) stained cerebellar sections from 21‐month‐old mice. (E) Cell density in the granular and molecular layers. Cell density was analyzed on pictures taken with a 40x (for granular layer) and a 20x (for molecular layer) objective. Cell numbers in 12 randomly picked 18 × 18 mm squares, three from each of four sections, were counted to obtain the density for each mouse. ***P < 0.0001 by *t*‐test; ns, not significant.

Previously we reported that *Immp2l* ^*−/−*^ (written as −/− thereafter) mice develop progressive ataxia starting from the age of 16 months (George *et al*., 2011). We suspect that ataxia in old mutant mice could result from impairment in the nervous system elements that maintain body balance and coordination. Mice with impairments in controlling body balance and coordination show a hind limb clasping response upon tail suspension (Lalonde & Strazielle, 2011). Consistently, young control and mutant mice show no significant difference in clasp response after tail suspension, whereas most mutant mice show clasp response at the age of 20 months (Table 1, Fig. 1A). No tremor is observed when they are at rest. Normal control mice and *Immp2l* ^*+/−*^ mice do not develop ataxia at the age of up to 30 months, the oldest age examined.

**Table 1:** Clasp response after tail suspension

	+/+	−/−	Fisher's test
Young (≤ 10 month old)	0/25	3/20	*P* = 0.08
Old (≥ 20 month old)	0/6	11/12	*P* = 0.0004

Numerators indicate the number of mice showing clasp response, and denominators indicate the numbers of mice tested.

Because the cerebellum plays an important role in maintaining balance, we examined whether old homozygous mutant mice have cerebellar abnormalities. The weight of cerebella from mutant mice of 21 months is significantly reduced (Fig. 1B), suggesting significant hypoplasia. Histologic analyses confirmed smaller cerebella from mutant mice (Fig. 1C).

More detailed studies showed that hypoplasia was present in the granular layer in −/− mutant mice (Fig. 1D). All 7 mutant mice showed reduced granule cell density after the age of 16 months, but none of the 5 age‐matched control mice did. Mean granule cell density in 21‐month‐old mice was 29.2 ± 0.3 cell/arbitrary area (*n* = 5) for +/+ mice, but 21.2 ± 0.8 cell/arbitrary area (*n* = 5) for −/− mice (Fig. 1E, *P* < 0.0001). Considering that cerebella from mutant mice are smaller, our data suggest that their total CGN number could be greatly decreased. Cerebella of 21‐month‐old +/− mice showed comparable histology to their age‐matched +/+ littermates. Hypoplasia was not observed in 3‐month‐old mutant mice, ruling out defective cerebellar development.

Hypoplasia seems to be specific for CGNs. We noticed no reduction of Purkinje cells in −/− mice old than 16 months. The cell density in the molecular layer of 21‐month‐old −/− mice (9.7 ± 0.5, *n* = 6) is also comparable to that of +/+ mice (9.2 ± 0.4, *n* = 6, *P* = 0.48) (Fig. 1E). Thus, CGN loss is the most obvious defect in old mutant mice showing ataxia and is associated with age‐dependent ataxia.

### The mitochondria‐targeted antioxidant SkQ1 ameliorates CGN degeneration in mutant mice

The mitochondria‐targeted antioxidant SkQ1 can reduce oxidative stress and elongate lifespan in animal studies (Antonenko *et al*., 2008; Shipounova *et al*., 2010; Anisimov *et al*., 2011; Skulachev, 2011). It is used to treat dry eye syndrome in Russia. Our previous work suggested that excessive mitochondrial superoxide generation in the mutants might underline the phenotypes in the non‐nervous system (Lu *et al*., 2008; George *et al*., 2011, 2012). We tested whether SkQ1 treatment could protect CGNs in mutant mice. Five +/+ and five −/− females were treated with 0.3 μm SkQ1 in drinking water (diluted 10^4^‐fold from concentrated solution prepared in 50% ethanol) from the age of 6 weeks. Three +/+ and three −/− treated mice survived the treatment and were sacrificed at the age of 21 months to examine neurodegeneration. Mice receiving tap water were used as untreated controls.

**Figure 2:**
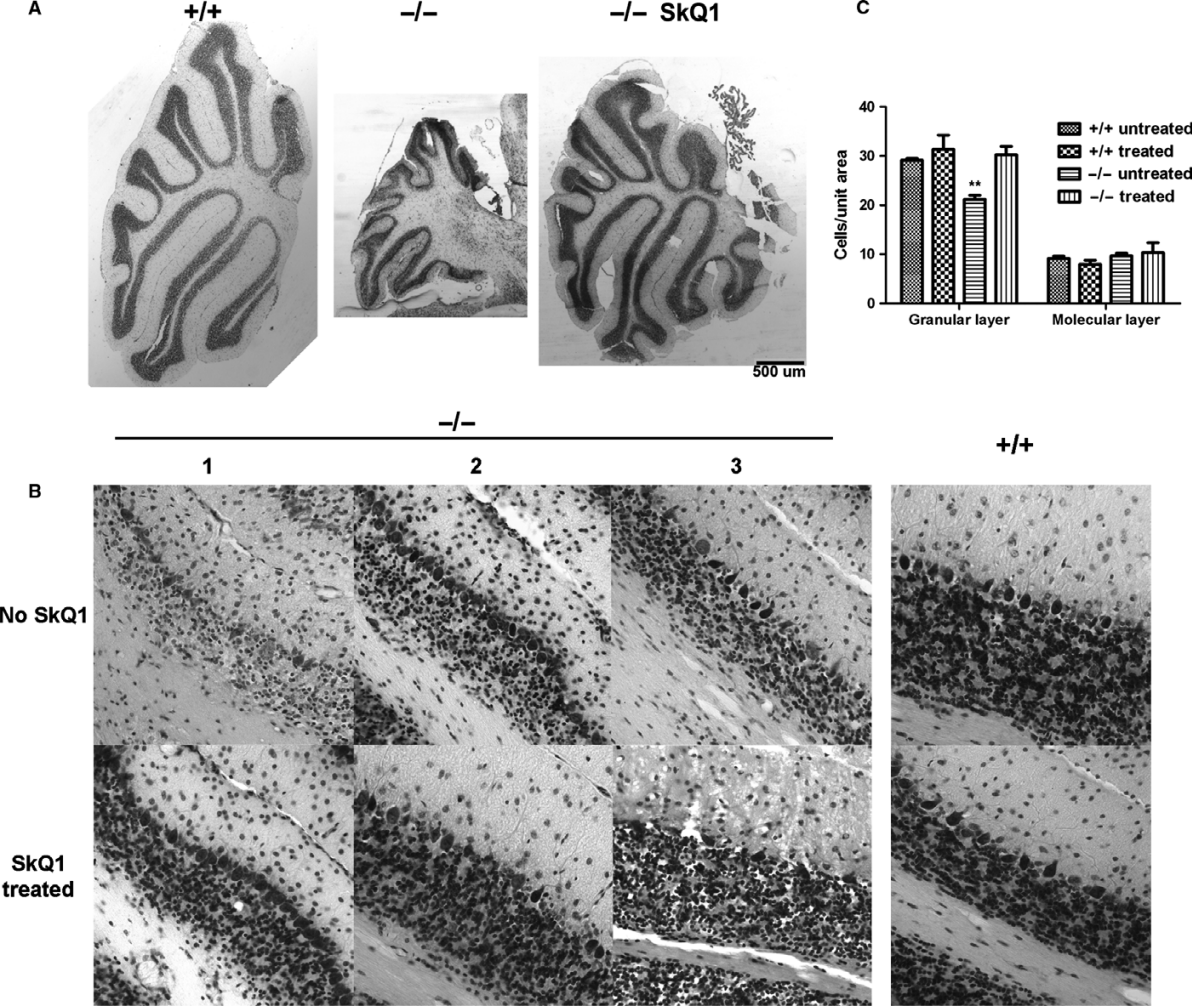
SkQ1 treatment protects CGNs in female mutant mice. (A) SkQ1 treatment restores cerebellar size of mutant mice. Sections were taken around the middle plane of sagittal cerebellar sections. (B) SkQ1 treatment increased cerebellar granule neuron density in mutant mice. Numbers 1‐3 indicate three different animals. All sections were of the same thickness and were processed in parallel. All animals were sacrificed at the age of 21 months. (C) Quantitative analyses of cerebellar cell density in control and mutant mice. *n* = 5 and 3, respectively, for SkQ1 untreated and treated mice. **Indicates *P* < 0.01 (compared with any other groups) by Tukey *post hoc* tests following ANOVA.

Whereas 100% untreated −/− female mice showed ataxia from the age of 16 months, none of the three SkQ1‐treated mutant mice showed ataxia even at euthanasia (21 months). Cerebella of SkQ1‐treated −/− mice were similar in size to those of control mice and significantly larger than those of untreated mutant mice (Fig. 2A). In addition, all three SkQ1‐treated −/− mice had increased granule cell density compared to untreated −/− mice (Fig. 2B). SkQ1‐treated −/− mice showed similar granular layer cell density as +/+ mice, but significantly higher granular layer cell density than untreated −/− mice (Fig. 2C). SkQ1 treated +/+ mice showed no difference in cerebellar size and granule cell density compared with untreated +/+ mice. Cell density in the molecular layer was similar between all groups.

### CGNs in old mutant mice were lost through apoptosis

**Figure 3:**
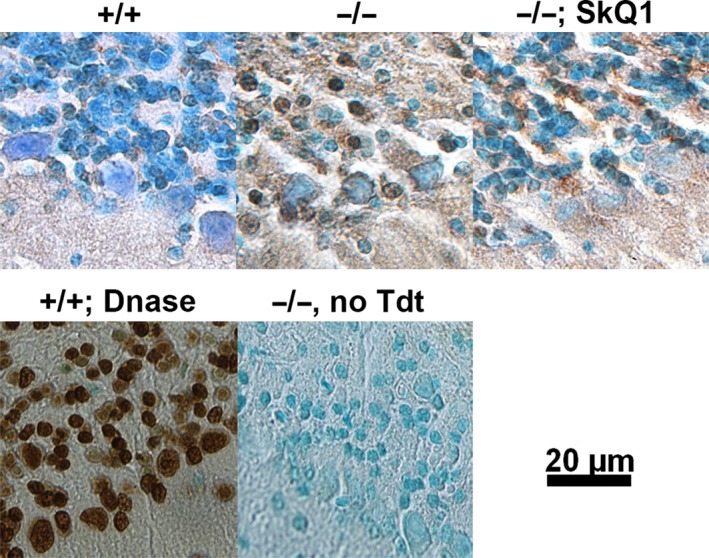
TUNEL assays detected apoptotic cells in old mutant mice. TUNEL‐positive granule cells (stained brown in the nuclei) were observed in 21‐month‐old mutant mice (Top, middle), but not in age‐matched normal control mice (Top, left), or SkQ1‐treated 21‐month‐old mutant mice (Top, right). The nuclei were counterstained by methyl green, which is less able to stain the TUNEL‐positive nuclei. DNase I was included during the staining for positive control, which made nearly all nuclei positively stained (bottom left). Terminal deoxynucleotidyl transferase (Tdt) was omitted for negative control, where no nuclei were positive. Brownish staining outside the nuclei indicated nonspecific signals.

To explore whether increased apoptosis in mutants explains CGN loss, we conducted TUNEL assays. No typical TUNEL‐positive cells were observed in 21‐month‐old +/+ mice (Fig. 3A, top row, left), whereas up to 25% of CGN nuclei were TUNEL‐positive in age‐matched −/− mice (Fig. 3A, top row, middle). Purkinje cell nuclei were not TUNEL‐positive. Consistent with no CGN loss in SkQ1 treated −/− mice, TUNEL‐positive CGNs (with brown nuclei) were not observed in these mice (Fig. 3A, top row, right). As a positive control, DNase I treatment during labeling made all cells positively labeled (Fig. 3A, bottom left). On the contrary, no positive cells were seen when terminal deoxynucleotidyl transferase was omitted (negative controls) (Fig. 3A, bottom right). No TUNEL‐positive CGNs were observed in 13‐month‐old −/− mice (data not shown). The data suggest that apoptosis contributed to CGN loss in old mutant mice.

### Old mutant mice had increased oxidative stress

**Figure 4:**
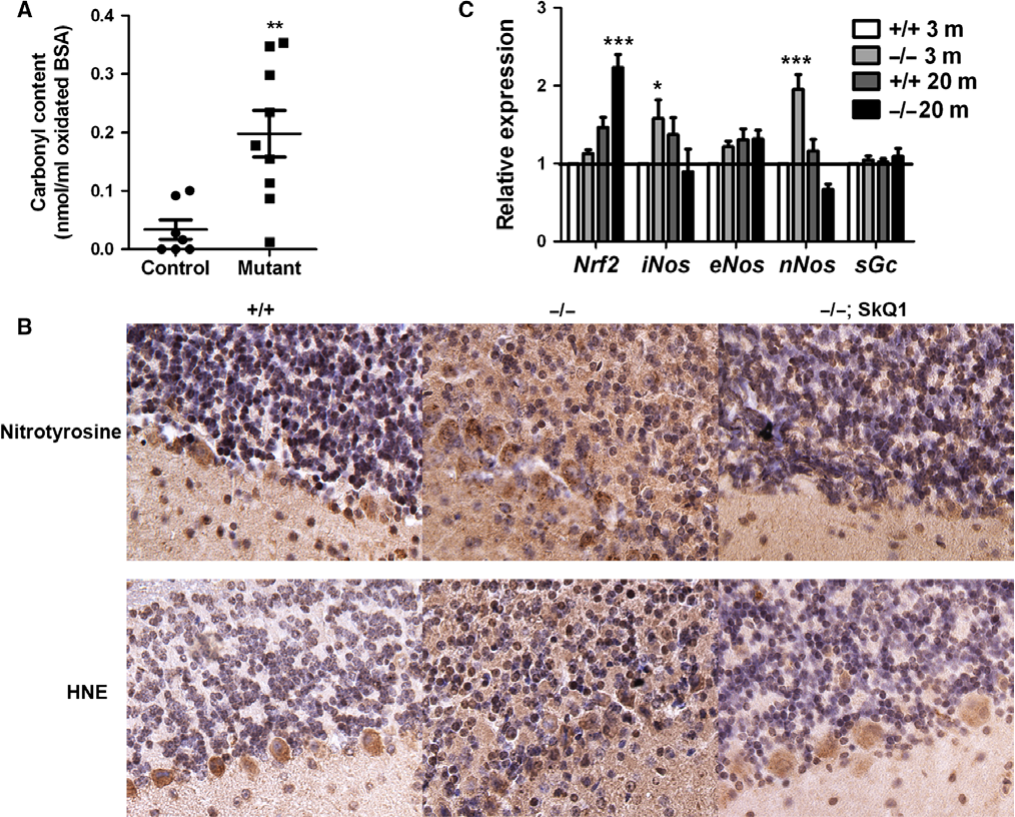
Increased oxidative stress in old mutant mice. (A) Serum carbonyl content. Each point indicates one mouse. **Indicates *P* < 0.01 by *t*‐test. (B) Immunohistochemical detection of HNE and tyrosine nitration modification of proteins in old mutant mice. All sections for the same antibody were put on the same glass slide for immunostaining. Negative controls without primary antibodies were all negative for DAB staining. In images, the granular layer is at the top and the molecular layer at the bottom. The nuclei were counterstained by hematoxylin (purple). (C) qRT–PCR analysis of cerebellar gene expression. Four animals were analyzed for each group. * and *** indicate *P* < 0.05 and *P* < 0.0001 by Bonferroni *post hoc* tests following ANOVA. For all panels, means ± SEM are shown.

To see whether old mutant mice have increased oxidative stress, we first compared serum carbonyl content in blood samples from 21‐month‐old control and mutant mice. Sera from −/− mice showed significantly increased carbonyl content compared to +/+ mice (Fig. 4A), suggesting that old mutant mice had increased systemic oxidative stress. For unknown reasons, SkQ1 treatment increased serum protein carbonyl content in both +/+ and −/− mice. Thus, protein carbonyl content appears to be an unsuitable oxidative stress index in SkQ1‐treated mice. When we attempted to compare the protein carbonyl content in cerebellar lysates by ELISA, only the lysates from two mutant mice (8 mutant and 11 control mice of 17–24 months assayed) showed protein carbonyl content above background.

To further examine whether oxidative stress in the cerebellum of mutant mice was increased, we used immunohistochemistry to compare the degree of 4‐hydroxynonenal (HNE) and nitration modification of proteins in the cerebellum. Both modifications were significantly increased in the cerebellum of 18‐month‐old (data not shown) and 21‐month‐old mutant mice (Fig. 4B, middle). This increase was not specific to any cell type, but occurred in all cerebellar areas of the mutant mice. Purkinje cells showed stronger signals for both modifications, even though they are not apparently affected in the mutants. Consistent with SkQ1 treatment protecting CGNs in mutant mice, SkQ1 treatment decreased HNE and nitration modification of proteins in mutant mice (Fig. 4B, right). Consistent with young mutant mice showing no CGN degeneration, HNE and nitration modification of proteins was not increased in the cerebella of 3‐month‐old mutant mice (Fig. S1).

### Nitric oxide synthase expression is increased in young mutant mice but not old mutant mice

Peroxynitrite, the product of superoxide and nitric oxide, is the major mediator of protein nitration in some cells (Galinanes & Matata, 2002). Our observation of increased protein nitration in old mutant mice is consistent with our previous observation of excessive superoxide generation from mitochondria of mutant mice (Lu *et al*., 2008). Physiological levels of nitric oxide are necessary for the survival of differentiated cerebellar granule cells (Ciani *et al*., 2002). nNOS is highly expressed in cerebellar granule neurons (Bredt *et al*., 1990; Bredt & Snyder, 1992). We previously reported that −/− mice show erectile dysfunction and decreased hypothalamic cGMP level (Lu *et al*., 2008; Han *et al*., 2013), both could result from negation of nitric oxide by mitochondrial superoxide. Quantitative RT–PCR revealed that *nNOS* and *iNOS* expression were increased in cerebella of young mutant mice, whereas *eNOS* expression was not significantly different between +/+ and −/− mice regardless of age (Fig. 4C). *nNOS* was slightly decreased in old mutant mice. Although *iNOS* was slightly increased in young mutant mice, the possibility of nitric oxide toxicity from *iNOS* overexpression is unlikely, because CGN loss is not seen in young mutant mice. Nitric oxide signaling occurs through soluble guanylyl cyclase (*sGc*); expression of mRNA coding for *sGc* was not different between control and mutant mice. Increased *nNos* expression in young mutant mice is consistent with the observation that cerebellar granule cells have high nNOS expression and that nitric oxide is necessary for survival of cerebellar granule cells (Ciani *et al*., 2002). However, our data excluded the possibility that increased production of nitric oxide contributed to increased peroxynitrite and thus protein nitration in old mutant mice.

### Old mutant mice had reduced SOD2 expression in the CGN but not Purkinje cells

**Figure 5:**
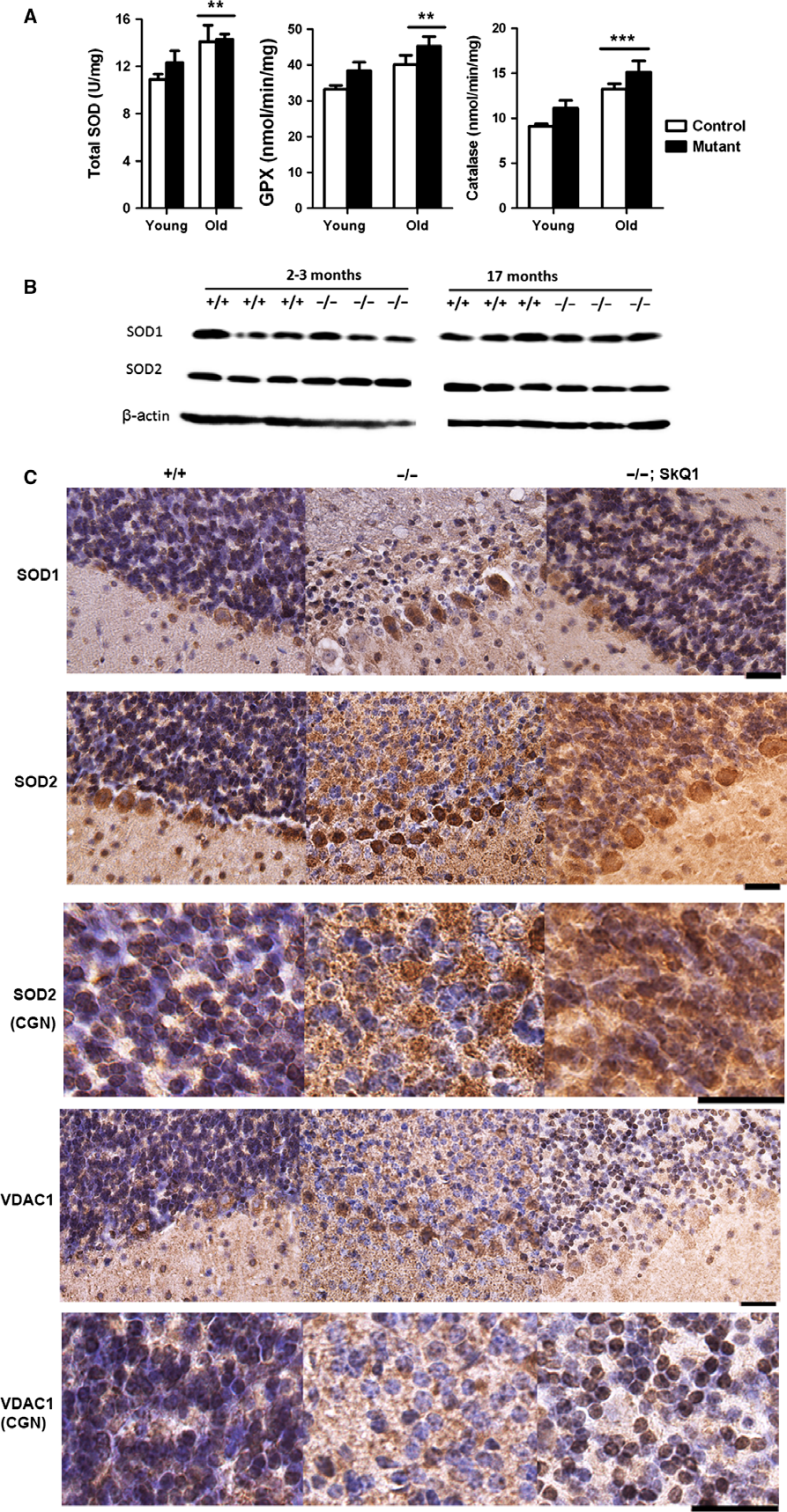
Comparison of antioxidant enzymes and mitochondrial marker VDAC1. (A) Total SOD, glutathione peroxidase, and catalase activity in cerebellar lysates. Young mice were 2–8 months, and old mice were 17–25 months. N for young +/+, young −/−, old +/+ and old −/− were 10, 7, 3, and 8, respectively. ** and *** indicate *P* < 0.01 and 0.0001 by Bonferroni *post hoc* tests following ANOVA. (B) Comparison of SOD1 and SOD2 expression in cerebella by Western blotting. (C) Comparison of SOD1, SOD2, and VDAC1 expression in cerebella by immunohistochemistry. All tissues were from 21‐month‐old mice. The nuclei were counterstained by hematoxylin (purple). In images, the granular layer is at the top and the molecular layer at the bottom. Scale bars: 20 μm.

To examine whether decreased antioxidant capacity explained increased oxidative stress in the cerebella of old mutant mice, we compared antioxidant enzyme activities in cerebellar lysates from control and mutant mice. Total superoxide dismutase (SOD) activity was similar regardless of age (Fig. 5A, left). Glutathione peroxidase (Fig. 5A, middle) and catalase (Fig. 5A, right) activity in cerebellar lysates were not reduced in mutant mice compared with age‐matched controls. Activity of all three enzymes was increased in cerebella of old mice compared with young mice, consistent with previous observations (Hussain *et al*., 1995). This observation is consistent with upregulated expression of *Nrf2* (nuclear factor, erythroid derived 2, like 2) mRNA in the cerebella of old mutant mice (Fig. 4C), a gene coding for a transcription factor regulating the expression of antioxidant genes (Vargas *et al*., 2008).

SOD1 and SOD2 are two cellular superoxide dismutases mainly localized in the cytoplasm and the mitochondria, respectively. Considering our previous observation of excessive mitochondrial superoxide generation in the mutants (Lu *et al*., 2008), we examine whether one of the two enzymes might be deficient in the mutant mice. Western blotting analysis of SOD1 and SOD2 expression in the cerebellum did not reveal deficiencies of either protein in mutant mice (Fig. 5B).

To test whether these proteins could be deficient in a specific cell type in the cerebella of mutant mice, we also measured expression of these proteins on cerebellar sections by immunohistochemistry. Purkinje cells showed stronger expression of the proteins than granule neurons (Fig. 5C). SOD1 expression was not significantly different when comparing the same cell type in control and mutant mice. However, SOD2 was significantly increased in Purkinje cells of old mutant mice compared to controls. In the granular layer of mutant mice, SOD2 signal was increased outside of granule neurons but decreased in granule neurons (Fig. 5C, third row). The signals outside the granule neurons were most likely also specific SOD2 signals, because the granular layer has neurites from Purkinje cells, Golgi cells and other neurons, which have mitochondria and thus SOD2. Increased SOD2 expression in Purkinje cells of old mutant mice could be a response to increased superoxide generation, because superoxide can induce SOD2 expression (Hu *et al*., 2005). Again, differential SOD2 expression in 3‐month‐old control and mutant mice was not as prominent as observed in old mice (Fig. S2). SkQ1 treatment did not prevent the increase of SOD2 outside the granule neurons, but prevented the decrease of SOD2 in the granule neurons (Fig. 5C, right). Thus, a decrease of SOD2 specifically in CGN cells correlates with CGN loss in the old mutant mice.

### CGNs of old mutant mice had reduced mitochondrial marker protein VDAC1

As SOD2 is a mitochondrial protein, we sought to determine whether reduced SOD2 expression in CGNs of old mutant mice reflects mitochondrial deficiency in mutant CGNs. We used immunohistochemistry to compare the expression of VDAC1, a mitochondrial protein widely used as a loading control for mitochondrial proteins. This voltage‐dependent anion channel, a mitochondrial outer membrane protein, is highly unlikely to be affected by IMMP2L deficiency in the mutants, because IMMP2L's substrates are inner membrane proteins, and so far only CYC1 and GPD2 are known IMMP2L substrates (Lu *et al*., 2008). Similar to SOD2, we found specific reduction of DVAC1 expression in granule neurons of old mutant mice (Fig. 5C). Unlike the upregulation of SOD2 outside the granule neurons in the granular layer of old mutant mice, VDAC1 was not upregulated outside granule neurons, suggesting that increased mitochondrial superoxide can up regulate SOD2 expression, but not VDAC1 expression or mitochondrial biogenesis. Differential expression of VDAC1 was not observed in 3‐month‐old control and mutant mice (Fig. S2). Consistent with no CGN degeneration in SkQ1‐treated mice, CGNs of SkQ1‐treated mice had normal VDAC1 expression (Fig. 5C). Thus, our data suggest that the quantity of mitochondria is deficient in CGNs of old mutant mice.

## Discussion

Here we report that *Immp2l* mutant mice show age‐dependent CGN degeneration. Based on our published and current data, we propose that the age‐dependent neurodegeneration observed in *Immp2l* mutant mice is caused by oxidative stress. Four lines of evidence support this conclusion. First, mutant mice had elevated levels of mitochondrial superoxide, which explains their phenotype of erectile dysfunction (Lu *et al*., 2008). Second, mitochondrial bioenergetic profiles in mutant mice are indistinguishable from those of normal control mice (Bharadwaj *et al*., 2014), excluding the possibility of mitochondrial energy deficiency. Third, protein carbonyl content, and HNE and nitration modification of proteins are increased in old mutant mice. And fourth, treatment with the antioxidant SkQ1 protected against neurodegeneration in old mutant mice and reduced HNE and nitration modification of proteins. To the best of our knowledge, this is the first observation of age‐dependent neurodegeneration in a model other than one expressing a known neurodegeneration‐causative human mutation.

Ataxia and CGN loss are seen in many patients with prion disease (Collinge *et al*., 1992; Faucheux *et al*., 2011) and in transgenic mice expressing a prion protein insertional mutation (Chiesa *et al*., 2000). Muzaimi *et al*. reported that the clinical prevalence of idiopathic late‐onset cerebellar ataxia is 8.4 per 100 000 persons, and the average age of onset is 53.8 years (Muzaimi *et al*., 2004). Determining the mechanism of granule cell degeneration will shed light on degeneration of other neuron types and neurodegeneration in other age‐related neurodegenerative diseases.

So far we have not observed neurodegeneration in other regions of the brain in old mutant mice. In the cerebellum, the Purkinje cells were apparently unaffected. SOD2 and VDAC1 both were reduced in CGNs of old mutant mice with CGN degeneration, and SOD2 and VDAC1 were restored in SkQ1‐treated mutant mice without CGN degeneration. VDAC1 deficiency in CGNs of old mutant mice indicates that mitochondrial content in CGNs of old mutant mice is decreased. At present, we are unsure whether SOD2 deficiency is the cause or the consequence of mitochondrial deficiency. However, mitochondrial deficiency is most likely the major reason why CGNs degenerate in old mutant mice. We observed stronger SOD1 and SOD2 expression in the Purkinje cells than in granule neurons, which could be one of the reasons why Purkinje cells appear resistant to the mutation.

**Figure 6:**
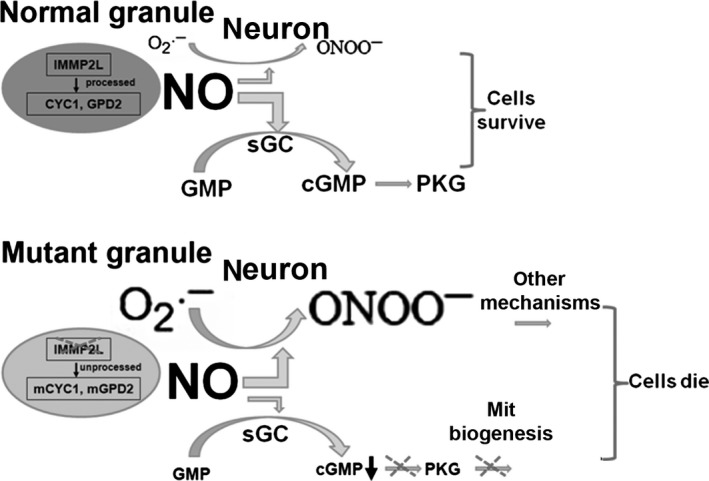
Proposed model for CGN degeneration in mutant mice. CGNs have high nNOS expression and depend on nitric oxide signaling for survival. CGNs of normal mice produce low levels of superoxide, and there is sufficient nitric oxide to support granule cell survival after superoxide and nitric oxide react to form peroxynitrite. CGNs of mutant mice produce increased levels of mitochondrial superoxide, which enhances peroxynitrite formation and reduces the bioavailability of nitric oxide. One consequence of reduced nitric oxide signaling is impaired mitochondrial biogenesis. SkQ1 treatment scavenges superoxide and protects CGNs in mutant mice.

The most likely explanation for CGN vulnerability in the mutants is the high nNOS expression in CGNs (Bredt *et al*., 1990; Bredt & Snyder, 1992) and the need for nitric oxide signaling for mature granule neuron survival (Pantazis *et al*., 1998; Bonthius *et al*., 2002; Ciani *et al*., 2002). In CGNs of normal mice, superoxide production and formation of peroxynitrite (product from a rapid reaction between superoxide and nitric oxide) are both low, and there is sufficient nitric oxide to support granule cell survival. However, in CGNs of mutant mice, increased mitochondrial superoxide generation enhances peroxynitrite formation (which is supported by increased protein nitration) and reduces the bioavailability of nitric oxide. Exactly how mitochondrial deficiency is caused in CGNs of old mutant mice is unclear and warrants further study. At least one of the consequences of reduced nitric oxide signaling is reduced mitochondrial biogenesis, because nitric oxide signaling is necessary for mitochondrial biogenesis (Nisoli *et al*., 2003). Either increased peroxynitrite production, reduced nitric oxide signaling, or both could contribute to mitochondrial deficiency and CGN degeneration (Fig. 6B). Thus, our observation of *nNOS* mRNA up‐regulation in cerebella of young mutants could represent a feedback response to increased negation of nitric oxide by superoxide.

Appropriate animal models to study oxidative stress in neurodegeneration are critically needed. Knockout of antioxidant enzymes, such as glutathione peroxidase, thioredoxin, and SOD2, causes embryonic/postnatal lethality. SOD1 deficiency increases oxidative stress, but does not cause neurodegeneration in the central nervous system. Therefore, the *Immp2l* mutant mouse model could be a useful model to elucidate the role of oxidative stress in neurodegeneration.

Although more research is needed to confirm the effects of the mitochondria‐targeted antioxidant SkQ1 on CGN degeneration, our preliminary data suggest that it could be highly effective. SkQ1 scavenges superoxide (Chistyakov *et al*., 2012), and SkQ1 is enriched in mitochondria. These two features may explain why SkQ1 is effective in protecting CGN degeneration in *Immp2l* mutant mice, because mitochondria in those mice generate elevated levels of superoxide due to abnormal processing of the mitochondrial inner membrane proteins CYC1 and GPD2 (Lu *et al*., 2008). Although SkQ1 protected against CGN degeneration in mutant mice, it had little effect on CGN density in normal control mice. These data suggest that the beneficial effects of antioxidants may only be apparent in individuals with oxidative stress. In the present study, we initiated SkQ1 treatment from the age of 6 weeks. Thus, it is unknown whether later treatment could also be effective. This information would be valuable, because clinical treatment of neurodegeneration is usually initiated only in its late stages.

In summary, we report here that *Immp2l* mutant mice show age‐dependent CGN degeneration and that the antioxidant SkQ1 protects against this effect. Our data suggest that oxidative stress is involved in CGN degeneration. The *Immp2l* mutant mouse model could be valuable in elucidating the role of oxidative stress in age‐associated neurodegeneration and in testing therapeutic strategies for that condition.

## Experimental procedures

### Animals

*Immp2l* ^*−/−*^ mice with the *Immp2l* ^*Tg(Tyr)979Ove*^ allele have been described previously (Lu *et al*., 2008). Mice were housed in the pathogen‐free animal facility at Wake Forest University Health Sciences. Experiments were conducted in accordance with the National Research Council publication Guide for Care and Use of Laboratory Animals and approved by the Institutional Animal Care and Use Committee of Wake Forest University Health Sciences. Mice were kept in microisolator cages with 12‐h light/dark cycles and were fed *ad libitum*.

### SkQ1 treatment

SkQ1 was a kind gift from Dr. Maxim Skulachev at Mitotech LLC, Russia. SkQ1 was delivered through drinking water, which was refreshed twice a week. Concentrated SkQ1 stock solution was prepared in 50% ethanol at a concentration of 30 mMol L^−1^ and kept at −20°C. Fresh SkQ1‐containing water was prepared by diluting the SkQ1 stock 10^4^‐fold with drinking water. SkQ1 treatment began when mice were 6 weeks old. To determine the concentration of SkQ1 in drinking water, daily water consumption was predetermined in adult female mice. Based on our observation that a 30‐g female mouse consumes about 5 ml water daily, the SkQ1 dosage was calculated at 50 nmol day^−1^ kg^−1^ body weight. This dosage elongated rodent lifespan in a previous publication (Anisimov *et al*., 2011). During treatment, four mice (2+/+ and 2−/− mice) were euthanized at the age of 10 months, due to dehydration caused by accidental water leakage. The surviving 3+/+ and 3−/− SkQ1‐treated female mice were euthanized at the age of 21 months to examine neurodegeneration. Mice receiving tap water were used as untreated controls.

### Cerebellar histology

Cerebellar tissues were fixed in 4% paraformaldehyde/PBS at 4°C overnight. Some of the tissues were embedded in OCT for cryosections, and some were dehydrated and embedded in paraffin. Samples of 5–8 μm (paraffin sections) or 10–15 μm (cryosections) were prepared for hematoxylin and eosin or Nissl staining.

### Immunohistochemistry

A 10‐μm‐thick paraffin sections from cerebella of control and mutant mice were put on the same glass slides to ensure similar staining conditions. Before primary antibody incubation, the deparaffinized and rehydrated sections were treated with 3% H_2_O_2_ for 10 min to remove endogenous peroxidase activity. After 30 min blocking, sections were incubated with respective primary antibodies at room temperature for 1 h. The antibodies used were rabbit anti‐SOD1 (#Ab13498, 1:500; Abcam, Cambridge, MA, USA), mouse anti‐SOD2 (#Sc133134, 1:200; Santa Cruz Biotechnology, Inc. Santa Cruz, CA, USA), mouse anti‐VDAC1 (Abcam, ab14734, 1:500), rabbit anti‐HNE‐protein adducts (# 393207, 1:200; EMD Millipore, Darmstadt, Germany), and rabbit antinitrotyrosine (# 06‐284, 1:200; EMD Millipore). After incubation with primary antibody, biotin conjugated anti‐rabbit or anti‐mouse secondary antibodies (Vector Laboratories, Burlingame, CA, USA) were diluted at 1:300 and incubated with the sections at room temperature for 1 h. Then, the sections were incubated with VECTASTAIN Elite ABC Reagent, R.T.U. (Vector Laboratories) for 30 min at room temperature. Signals were visualized with ImmPACT DAB peroxidase (HRP) substrate (Vector Laboratories). Sections from different types of mice and treatments on the same slide were stained by identical methods. Negative controls without primary antibodies were included.

Images were acquired with an Axio M1 microscope equipped with an AxioCam MRc digital camera (Carl Zeiss, Thornwood, NY, USA). Different images were assembled into one file with Adobe Photoshop, with necessary resizing, rotation, and cropping.

### Cerebellar cell density quantification

Cerebellar cell density was determined on 14‐μm‐thick stained sections. Four nonsuccessive sections were stained for each mouse. Three images were taken from each section under a Leica MM4000B microscope equipped with a Retiga‐2000DC digital camera. 40× and 20× objectives were used to count CGNs and cells in the molecular layers, respectively. The images were opened with Photoshop. Three 18 mm^2^ squares were drawn randomly in the granular layer of images taken under 40× objective to determine numbers of CGN/square. Similarly, three 18 mm^2^ squares were drawn randomly in the molecular layer of images taken under 20× objective to determine cell numbers/square for cells in the molecular layer. The averages of four sections from each mouse were used to determine the respective cell density for that mouse.

### TUNEL assays

The TACS 2 TdT DAB kit (Trevigen Inc., Gaithersburg, MD, USA) was used to detect apoptotic cells in sections. TUNEL assays were performed according to the manufacturer's instructions.

### Antioxidant enzyme activity assays

Assay kits from Cayman Chemical Company (Ann Arbor, MI, USA) were used to determine superoxide dismutase (Item # 706002), catalase (Item # 707002), and glutathione peroxidase (Item # 703102) activities in cerebellar lysates. Protein levels were estimated using a Pierce^™^ BCA Protein Assay Kit (Item # 23225; Thermo Fisher Scientific Waltham, MA, USA ). Superoxide dismutase activity is expressed as U mg^−1^ protein. Catalase activity is expressed as nmol formaldehyde formed per minute per mg protein. Glutathione peroxidase activity is expressed as nmol NADPH oxidized per minute per mg protein.

### Western blotting assays

Cerebella from 2 to 3 months (no CGN degeneration) and 17 months (with CGN degeneration) were collected and snap frozen in liquid nitrogen. They were stored at −80°C until analyzed. Cerebellar tissues were lysed in RIPA buffer with protease inhibitors (0.5 mm PMSF and 1× Complete Protease Inhibitor Cocktail, Roche Diagnostics Corporation, Indianapolis, IN, USA), and the extracts were subjected to SDS‐PAGE and Western blotting. Anti‐β‐actin antibody was from Sigma (#A5441, 1:5000; St Louis, MO, USA). SOD1 and SOD2 antibodies were the same as those for immunohistochemistry but used at 1:2000 and 1:1000, respectively. Horseradish peroxidase (HRP)‐conjugated secondary antibodies (1:1000) were purchased from Pierce (Rockford, IL, USA). Chemiluminescent reagents from Pierce were used to visualize the protein signals using an LAS‐3000 imaging system (GE Healthcare Bio‐Sciences, Pittsburgh, PA, USA).

### Quantitative RT–PCR (qRT–PCR) assays

Total RNA was extracted from cerebellar tissues with RNeasy Mini Kit (Qiagen, Valencia, CA, USA) following the manufacturer's instructions. Residual genomic DNA was removed by DNase treatment with a kit from Qiagen. Reverse transcription was performed using a High Capacity Reverse Transcription Kit (Life Technologies).

Real‐time PCR was performed on a 7300 real‐time PCR system (Thermo Fisher Scientific). *Ppib* (*PpibF*: TCGTCTTTGGACTCTTTGGAA, and *PpibR*: AGCGCTCACCATAGATGCTC) was used an internal control. Primers for genes detected were as follows: eNOSF (CCTTCCGCTACCAGCCAGA) and eNOSR (CAGAGATCTTCACTGCATTGGCTA) for *Nos3*, nNOSF (TCCTAAATCCAGCCGATCGA) and nNOSR (TCATGGTTGCCAGGGAAGAC) for *Nos1*, iNOSF (AGAGAGATCCGATTTAGAGTCTTGGT) and iNOSR (TGACCCGTGAAGCCATGAC) or *Nos2*, Nrf2F (CTTTAGTCAGCGACAGAAGGAC) and Nrf2R (AGGCATCTTGTTTGGGAATGTG) for *Nrf2*, sGCF (CTGCTGGTGATCCGCAATTAT) and sGCR (GATGGTATCATAGCCAGACTCCT) for *Gucy1b3* coding for soluble guanylate cyclase 1 beta. All primer pairs spanned introns to minimize the possibility of amplification from genomic DNA. SYBR Green PCR Master Mix (Applied Biosystems) was used for real‐time PCR. After the PCR amplification, a dissociation program was run and the amplified product was analyzed by electrophoresis to verify the specificity of the amplification. Relative levels of gene expression were calculated using the *ΔΔ*CT method. Equal amounts of cDNA from five mice in each group were pooled to serve as a template for real‐time PCR. Three independent experiments were performed with each experiment carried out in triplicate. Results were presented as mean ± SEM.

### ELISA for protein carbonyl content

Serum samples were diluted by 4900‐fold for protein carbonyl content assays, and assays were carried out using a kit from Cell Biolabs, Inc. (San Diego, CA, USA) according to the manufacturer's instructions. To assay protein carbonyl content in cerebellar lysates, contaminating genomic DNA (which makes the reading erroneously high) was removed by adding 0.5% polyethyleneimine, and centrifuging the samples at 6000 *g* at 4°C for 10 min before the samples were assayed.

### Statistical analysis

graphpad prism (GraphPad Software, Inc. La Jolla, CA, USA) software was used for statistical analyses. *T*‐tests were used to compare the averages of two groups. In cases of more than two groups or one factor, ANOVA was performed followed by Tukey or Bonferroni *post hoc* tests. *P* < 0.05 was regarded as statistically significant.

## Author contributions

Chunlian Liu and Xue Li processed and analyzed the samples. Baisong Lu designed the project, maintained the animals, analyzed the data, and wrote the manuscript.

## Funding info

No funding information provided.

## Conflict of interest

None declared.

## Supporting information


**Fig. S1** HNE and nitrotyrosine modification of proteins in young mice. Sections from control and mutant mice were put on the same slide for immunostaining. Color development time was slightly different than for sections of old mice; thus, these images are not directly comparable with images from old mice (Fig. 4B).
**Fig. S2** SDO1, SOD2 and VDAC1 expression in cerebella of young mice. Sections from control and mutant mice were put on the same slide for immunostaining. Color development time was slightly different than for sections of old mice, thus, these images are not directly comparable with images from old mice (Fig. 5C).
