# Supplementary material for: The Immp2l mutation causes age‐dependent degeneration of cerebellar granule neurons prevented by antioxidant treatment
Source: Aging Cell. 2015 Nov 30;15(1):167–76. doi: 10.1111/acel.12426 (PMC4717271; doi:10.1111/acel.12426)

Fig.S1. HNE and nitrotyrosine modification of proteins in young mice. Sections from control and mutant mice were put on the same slide for immunostaining. Color development time was slightly different than for sections of old mice; thus, these images are not directly comparable with images from old mice (**Fig.4B**).

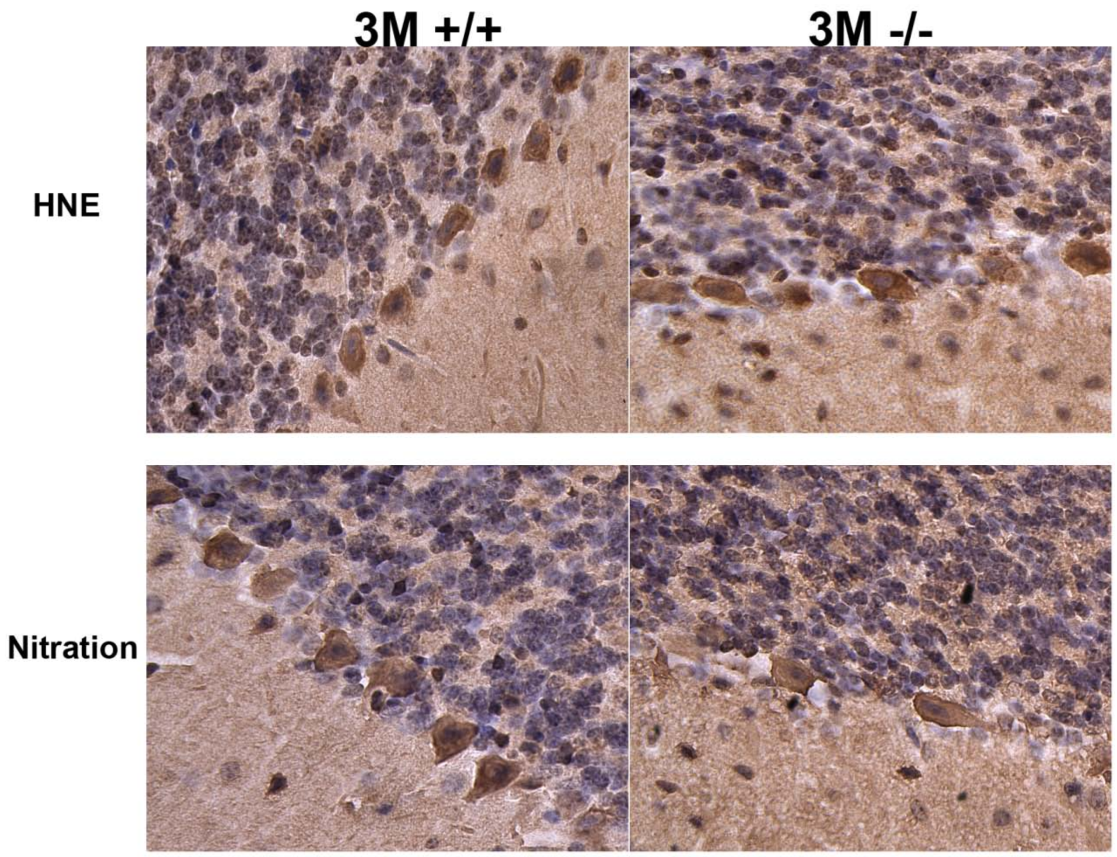

Fig.S2. SDO1, SOD2 and VDAC1 expression in cerebella of young mice. Sections from control and mutant mice were put on the same slide for immunostaining. Color development time was slightly different than for sections of old mice, thus, these images are not directly comparable with images from old mice (**Fig.5C**).

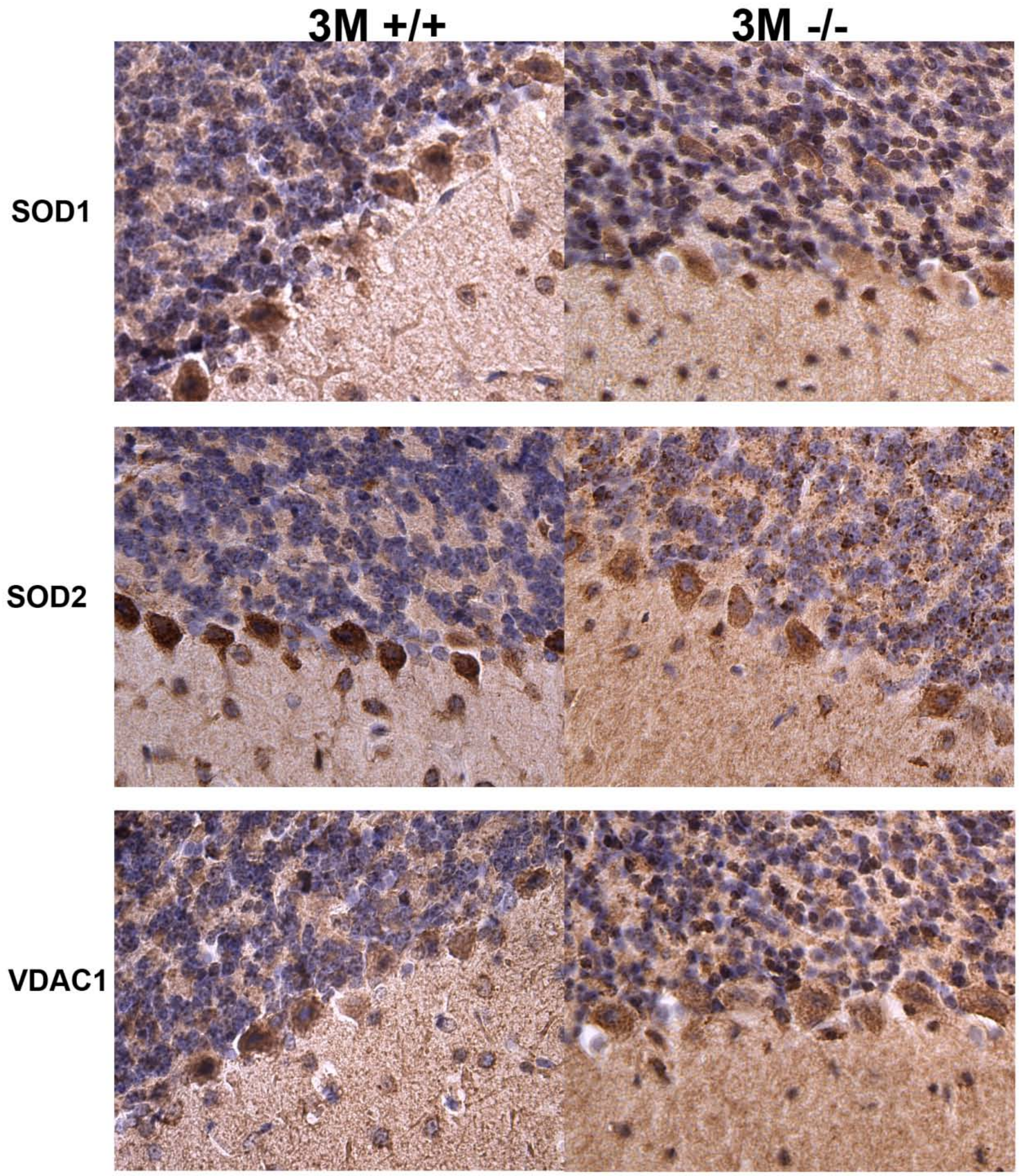

Supplement: Supplementary file 1 — Fig. S1 HNE and nitrotyrosine modification of proteins in young mice. Sections from control and mutant mice were put on the same slide for immunostaining. Color development time was slightly different than for sections of old mice; thus, these images are not directly comparable with images from old mice (Fig. 4B). Fig. S2 SDO1, SOD2 and VDAC1 expression in cerebella of young mice. Sections from control and mutant mice were put on the same slide for immunostaining. Color development time was slightly different than for sections of old mice, thus, these images are not directly comparable with images from old mice (Fig. 5C). [file ACEL-15-167-s001.pdf]
